# Enhancement of physiology via adaptive transcription

**DOI:** 10.1007/s00424-024-03037-5

**Published:** 2024-11-01

**Authors:** Thomas Lissek

**Affiliations:** Interdisciplinary Center for Neurosciences, Heidelberg University, Im Neuenheimer Feld 366, 69120 Heidelberg, Germany

**Keywords:** Enhancement, Physiology, Transcription, Adaptation, Brain, Muscle

## Abstract

The enhancement of complex physiological functions such as cognition and exercise performance in healthy individuals represents a challenging goal. Adaptive transcription programs that are naturally activated in animals to mediate cellular plasticity in response to stimulation can be leveraged to enhance physiological function above wild-type levels in young organisms and counteract complex functional decline in aging. In processes such as learning and memory and exercise-dependent muscle remodeling, a relatively small number of molecules such as certain stimulus-responsive transcription factors and immediate early genes coordinate widespread changes in cellular physiology. Adaptive transcription can be targeted by various methods including pharmaceutical compounds and gene transfer technologies. Important problems for leveraging adaptive transcription programs for physiological enhancement include a better understanding of their dynamical organization, more precise methods to influence the underlying molecular components, and the integration of adaptive transcription into multi-scale physiological enhancement concepts.

## Introduction

The improvement of physiological function in healthy individuals can enable the maintenance of health and the enhancement of resilience in young individuals as well as the compensation of health decline in old age. At human frontiers such as space exploration, enhancement of physiology could enable better performance under adverse environmental conditions. As many biological processes in mammals likely already operate near optimal levels and functional increases oftentimes require complex coordination of adaptation in several tissues, the genuine improvement of organism function by exogenous means is highly challenging. Yet, there exist several natural processes such as learning and memory, immune system plasticity, and exercise-induced increases in athletic capacity through which an animal increases its performance at certain tasks over the long-term. Based on this insight, adaptation mechanisms that are employed by organisms in these processes could potentially be targeted to achieve physiological enhancement.

A core mechanism for orchestrating the underlying cellular changes in natural complex adaptation processes such as learning is adaptive transcription involving transcription factors such as cAMP response element binding protein (CREB), serum response factor (SRF), and myocyte enhancer factor 2 (MEF2) and immediate early genes (IEGs) such as Fos oncogene (Fos) and early growth response 1 (Egr1). Adaptive transcription here is defined as transcriptional changes that are induced by acute stimulation and which coordinate adaptive changes in cellular physiology ranging from hours to days to weeks. It is thus distinguished from constitutive transcription programs (e.g., housekeeping genes) or less dynamical transcriptional changes (e.g., inducible but afterwards stable expression of cell-type defining genes during development). Adaptive transcription is highly plastic (e.g., different inputs lead to different gene induction patterns) and dynamic (e.g., it can be induced and shut-off on the minute timescale) [44].

Not only do animals use these gene programs for adaptation, but activation of adaptive transcription via complex measures has been shown to induce younger organism phenotypes, to increase organism resilience, and to protect against aging-related functional decline [42]. Gaining control over and leveraging adaptive transcription programs might hence be a valuable strategy for general improvement of physiological function and for counteracting aging-related disorders. By boosting these ubiquitous cellular adaptation mechanisms in a body-wide fashion, one might enhance the body’s ability to reprogram itself optimally to meet certain demands and challenges, a strategy termed here “adaptation-based enhancement.” The present work reviews and analyzes how adaptive transcription components can be targeted exogenously to improve complex physiological functions in healthy individuals beyond wild-type levels.

## Mechanisms for enhancing physiological function

### The problem of improving physiological function

Improving biological function is difficult for several reasons. The first is that mammalian organisms in general and humans in particular are highly complex with many interconnected components at various scales and most functional improvements are accompanied by changes in several organ systems throughout the body (e.g., exercise-induced adaptations in muscular tissue [22] and the cardiovascular system [33]) and interactions between different organ systems (e.g., between skeletal muscle and other organ systems [83]). Similarly, aging-related decline in body function is complex (e.g., involving sarcopenia, metabolic dysfunction, cognitive decline, and immune dysfunction), necessitating either the application of several interventions at once to counteract aging or a universal strategy that identifies targets that can be leveraged in several tissues simultaneously. But not only are organisms complex, they adapt and actively counteract modification from the outside. This is why many pharmacological treatments (oftentimes targeting a single or a few molecules) are either ineffective in the long-term as the body works against the signaling disturbance through homeostatic mechanisms or they induce substantial side-effects if the dose has to be increased to levels that the body cannot compensate for. In addition, many basic cellular processes most likely already operate near optimal levels, as evolution has had a long time to optimize them. A shift in any direction might entail trade-offs that degrade performance of the overall the system.

In spite of all of these difficulties, there are clear examples of inducible physiological improvements and anti-aging interventions in humans [42]. Usually, these are induced by complex environmental stimuli in the form of physical and cognitive exercise, as well as time-restricted nutritional scarcity. Examples include muscle strength and endurance increases with repeated physical exercise and mastering complex skills such as learning an instrument or a new language. In these examples, performance can oftentimes be increased drastically given sufficient time and without notable side-effects. Additionally, it has been shown that complex environmental stimulation can protect against aging-related decline and that it can increase health and resilience in elderly individuals [39, 48, 55, 60, 63, 97].

### Adaptive transcription mediates health improvements induced by natural stimuli and artificial interventions

Previous work has identified adaptive transcription to be a central mediator for the induction of younger phenotypes through cognitive and physical exercise, as well as dietary restriction and artificial interventions such as parabiosis [42]. For instance, CREB mediates learning and memory [9, 31, 37, 54], caloric restriction-induced cognitive effects in aged animals [27], and rejuvenation through blood transfer from young to old animals [94]. SRF is involved in memory formation [23] and mediates the rejuvenation effects of cerebrospinal fluid transfusion from young into old animals [34]. Building on the role of adaptive transcription in mediating inducible health benefits, the central question of the present work is whether it can be directly targeted to improve organism function and promote health. In the following section, molecules that demonstrate this principle will be discussed.

## Targeting adaptive transcription to enhance performance, resilience, and health

### Cognition and brain health

**Fig. 1:**
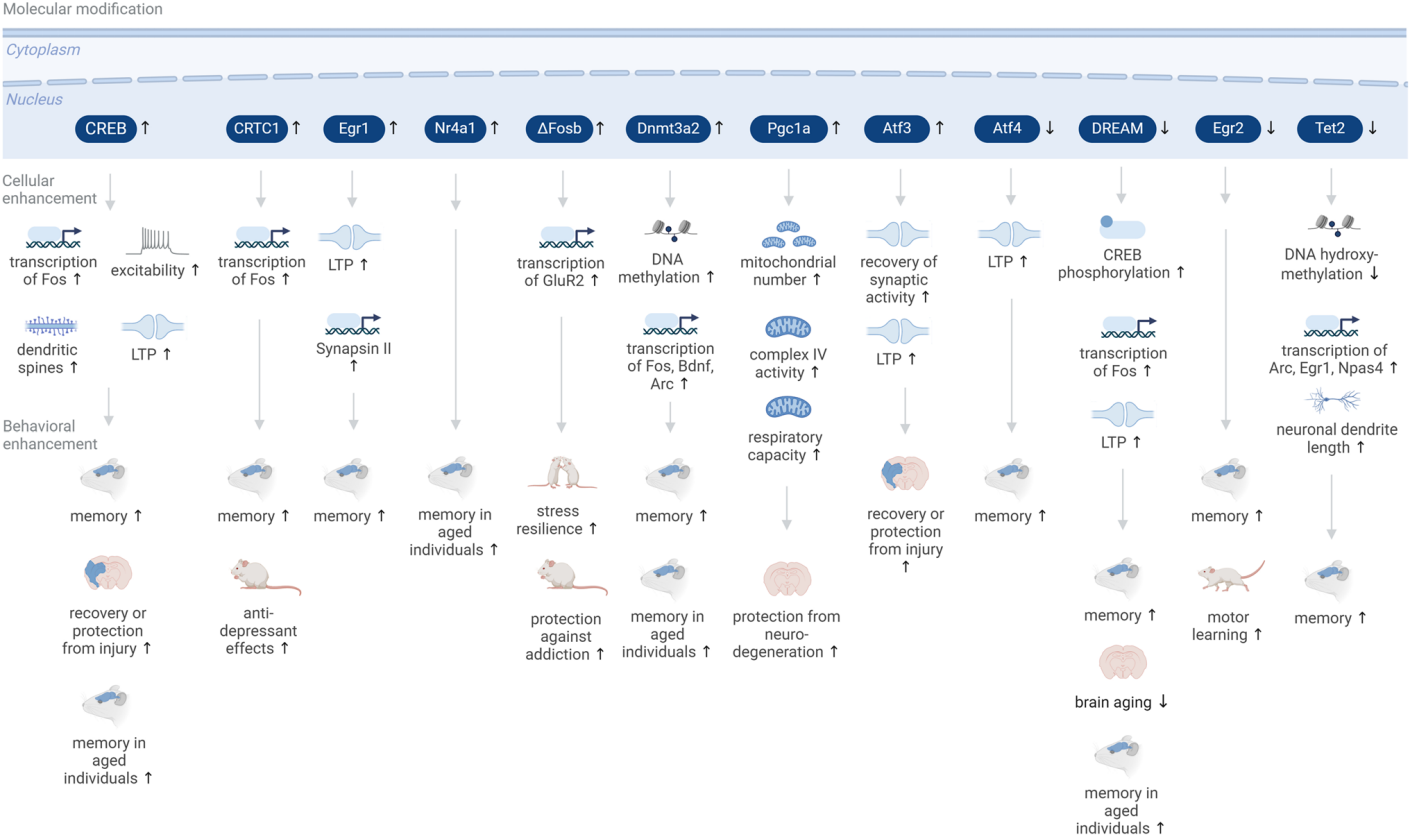
Enhancement of cognitive function through targeting adaptive transcription in the brain. Interventions targeting adaptive transcription mediators in neurons in the brain impact diverse cellular processes including activity-dependent transcription, synaptic plasticity, mitochondrial function, DNA methylation, and cellular morphological remodeling. At the behavioral level, these interventions lead to improvements in memory function, resilience against damage and against behavioral dysfunction and to a compensation of aging-related functional decline. Upward arrows for the molecular interventions denote an increase in the expression or activity of molecules; downward arrows denote a downregulation

Important domains for improving organism health are those of cognitive processes (e.g., learning and memory and resilience to stress) and brain health (e.g., resilience against neurodegeneration) (Fig. 1).

Enhancing CREB activity through transgenic expression of a dominant active CREB mutant in the mouse forebrain leads to enhanced expression of Fos in the CA1 hippocampal region and the amygdala (basal as well as behavior-induced), to increased long-term potentiation (LTP) in CA1 neurons, and to increased performance in contextual fear conditioning and water maze learning paradigms [87]. Similarly, CREB overexpression in the rat hippocampus via Herpes simplex virus (HSV) leads to facilitated place learning [10]. Viral expression of CREB-Y134F in either the hippocampal CA1 or DG areas in mice increases memory performance in contextual fear conditioning [78] and viral overexpression of CREB in the dorsal hippocampus of mice lowers the threshold for memory formation and induces spatial memory in training paradigms that do not induce memory in wild-type controls [81]. Viral CREB overexpression in the auditory thalamus of mice increases auditory conditioned fear memory [32]. With regard to aging, CREB overexpression in the dorsal CA1 via an adeno-associated virus (AAV) is able to enhance memory performance in a water maze paradigm in aged rats [102]. Viral overexpression of CREB also enhances recovery of neural circuit and motor function in mice after stroke [14]. At the cellular level, increased CREB activity or levels lead to changes in neuronal excitability [30, 95, 102, 109] and synaptic function [5, 30] as well as to increased spine density [82]. Viral overexpression of the CREB coactivator CREB-regulated transcription coactivator 1 (CRTC1) in the hippocampus leads to enhanced Fos induction in learning paradigms and improved memory [80] as well as antidepressant effects [66]. Inducible inhibition of activating transcription factor 4 (ATF4) via transgenic expression of its inhibitor AZIP in the forebrain of mice leads to enhanced LTP and improved memory performance in the Morris water maze [16]. In neurons, the transcriptional repressor DREAM negatively regulates immediate early gene induction and memory [61] and its in vivo transgenic deletion enhances CREB signaling and Fos induction, improves memory performance, and slows brain aging [25]. Reductions in the levels of the DNA-demethylating enzyme Tet methylcytosine dioxygenase 2 (TET2) result in differential gene methylation patterns, enhanced neuronal IEG induction, increased dendrite length, and enhanced memory performance [74].

At the level of activity-regulated genes, viral overexpression of the transcription factor deltaFosb in the nucleus accumbens of mice induces resilience against the effects of chronic social isolation stress, possibly through differential regulation of the AMPA receptor subunit GluR2 [93], and protects against addiction phenotypes [107]. Inducible transgenic Egr1 overexpression in the forebrain of mice enhances spatial memory and increases LTP in the dentate gyrus of the hippocampus [71]. Transgenic Egr2-deficiency in mice leads to an improvement in motor learning and object recognition memory [72]. Overexpression of the adaptive transcription factors nuclear receptor 4a1 (Nr4a1) and Nr4a2 in the mouse hippocampus via AAV leads to memory improvements in aged mice [38]. A gene that connects adaptive transcription to epigenetic remodeling is the activity-regulated DNA-methyltransferase Dnmt3a2. AAV-mediated overexpression of Dnmt3a2 in the hippocampus increases memory performance in aged mice [67] and young adult mice [68]. Dntm3a2 regulates IEG expression since its knockdown decreases induction of activity-regulated cytoskeleton-associated protein (Arc) and brain-derived neurotrophic factor (Bdnf) in neurons in vitro [67] and its overexpression increases expression of Fos and Arc in vivo [68]. Overexpression of several IEGs including the transcription factors neuronal PAS domain protein 4 (Npas4) and activating transcription factor 3 (Atf3) in the mouse hippocampus via AAVs protects against stroke-induced neuronal damage [105]. Similarly, transgenic overexpression of peroxisome proliferator-activated receptor gamma coactivator 1-alpha (Pgc1a) in mice in vivo leads to an increased number of neuronal mitochondria, enhanced complex IV activity, and increased respiratory capacity and protects against autoimmunity-mediated neurodegeneration [79]. Infusion of BDNF into the hippocampus of mice enhances memory persistence [7].

**Fig. 2:**
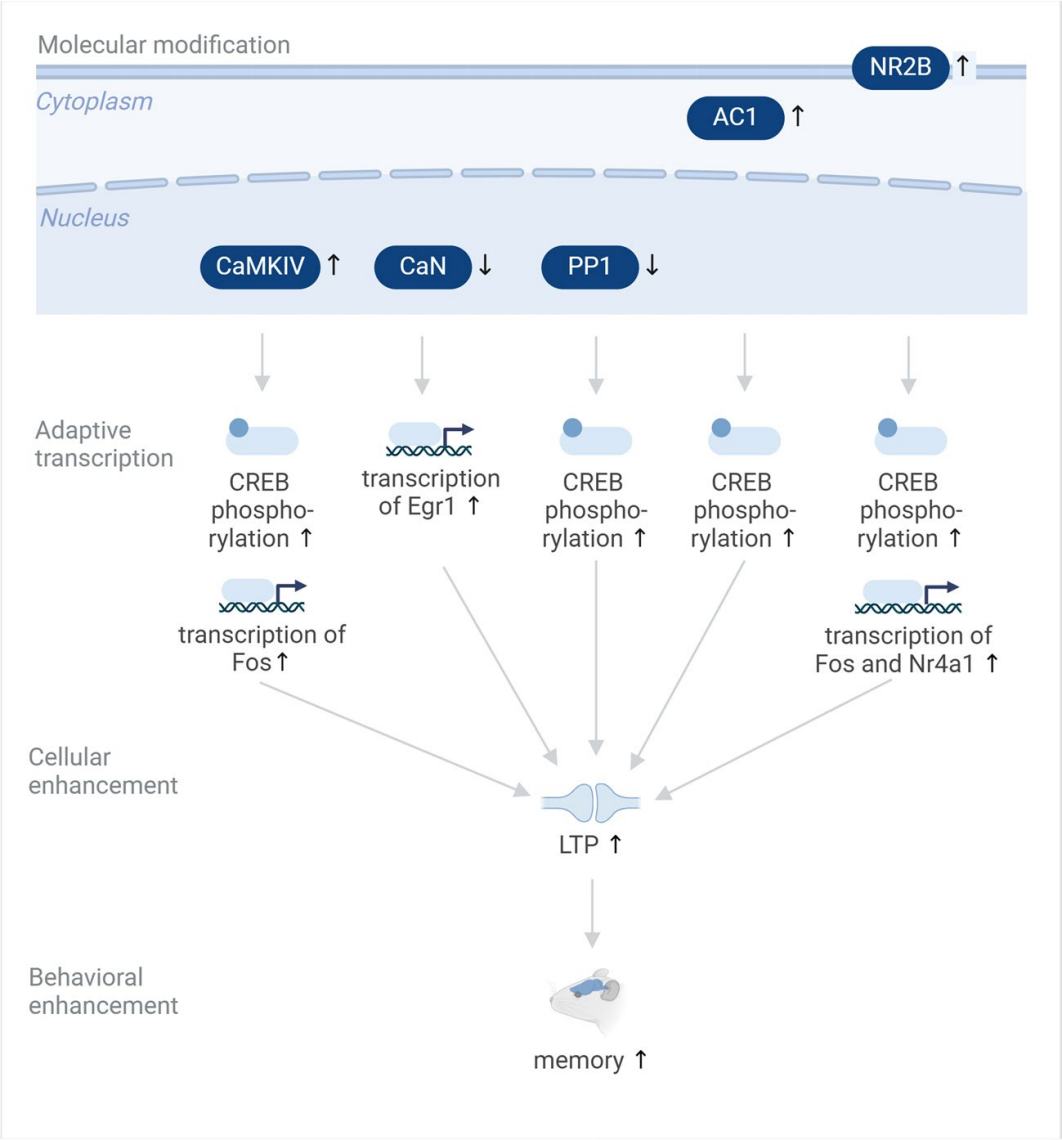
Enhancement of cognitive function through targeting upstream regulators of adaptive transcription in the brain. Upstream regulators of adaptive transcription including membrane receptors and cytosolic and nuclear enzymes can be targeted to enhance adaptive transcription in the brain with subsequent effects on cellular physiology and behavior. Upward arrows for the molecular interventions denote an increase in the expression or activity of molecules; downward arrows denote a downregulation

Previous studies have also shown that it is possible to achieve cognitive enhancements through overexpression of upstream regulators of adaptive transcription (Fig. 2). Transgenic overexpression of the NMDA receptor (NMDAR) subunit NR2B in the forebrain of mice leads to stronger electrophysiological NMDAR responses and enhanced memory [89], as well as increased memory performance in aged animals [13]. Forebrain NR2B overexpression also leads to increased prefrontal cortex LTP and improved working memory in transgenic mice [19], as well as increased CREB phosphorylation and transcription of Fos and Nr4a1 [40]. Transgenic overexpression of calcium/calmodulin-dependent protein kinase type IV (CaMKIV), a critical positive regulator of CREB activity and IEG induction, leads to enhanced CREB phosphorylation, increased hippocampal LTP, and increased memory performance in young adult and aged mice [26]. Another CREB activator is protein kinase A (PKA), which itself is activated by cAMP. Transgenic overexpression of Adcy1, which encodes the cAMP-producing enzyme adenylyl cyclase 1 (AC1), enhances CREB phosphorylation, LTP, and memory performance [99]. Inducible transgenic expression of a calcineurin inhibitor (i.e., the autoinhibitory domain in the C-terminus of CNAalpha) in the mouse brain enhances paired-pulse facilitation and LTP and improves memory performance in novel object and spatial learning paradigms [53]. CaN inhibition also increases Egr1 transcript levels and enhances the formation of aversive memories and their resistance to extinction [6]. Transgenic expression of an inhibitor of protein phosphatase 1 (PP1) increases phosphorylation of CREB and CaMKII, enhances learning performance and memory persistence in mice [28], enhances LTP, and differentially changes hippocampal synaptic plasticity [35].

### Muscle function

**Fig. 3:**
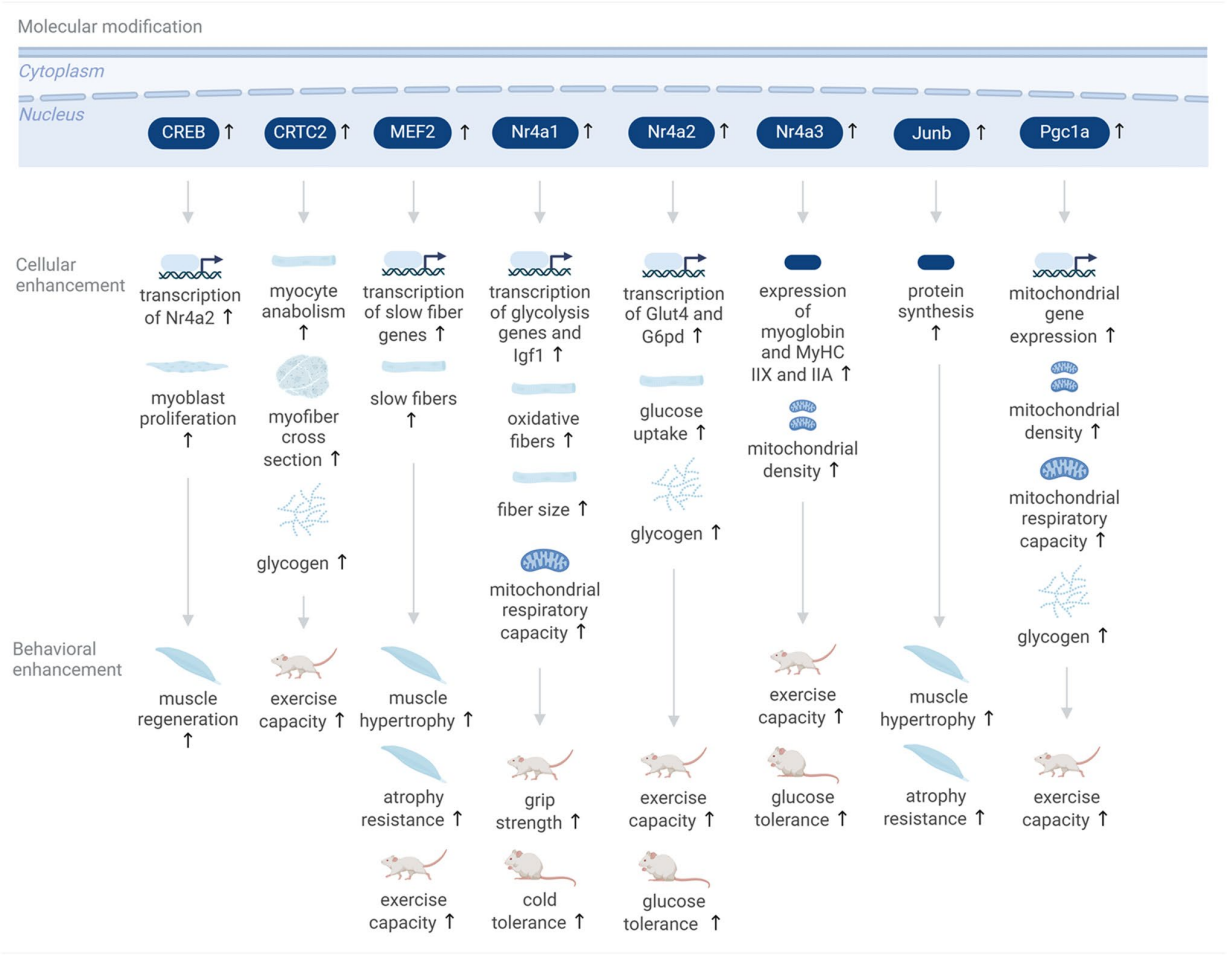
Enhancement of muscle function and exercise capacity through targeting adaptive transcription in skeletal muscle. Targeting adaptive transcription in skeletal muscle cells leads to changes in cellular physiology including differential transcription, altered myoprotein expression, cell architecture modifications, changes in glycogen and fatty acid content, and changes in mitochondrial function. These effects translate to improved muscle regeneration, increased exercise capacity, muscle hypertrophy and muscle atrophy resistance among others. Upward arrows for the molecular interventions denote an increase in the expression or activity of molecules; downward arrows denote a downregulation

Several adaptive transcription components have been shown to improve aspects of muscle function and related organism physiology when overexpressed (Fig. 3).

Whole body transgenic expression of the dominant active CREB mutant CREB-Y134F increases Nr4a2 transcription after cAMP-mediated stimulation in vitro, enhances myoblast proliferation, and improves muscle regeneration in mice in vivo [86]. Transgenic overexpression of the CREB coactivator CRTC2 in skeletal muscles of mice in vivo leads to an anabolic state in muscle cells, higher myofiber cross-section area, higher triglyceride and glycogen content in myocytes as well as increased exercise performance and lowered lactate levels after exercise [11]. Transfection of a constitutively active MEF2 mutant into rat skeletal muscles in vivo leads to muscle hypertrophy and counteracts atrophy after denervation [64]. Transgenic expression of a constitutively active MEF2 mutant in skeletal muscle in mice increases slow-fiber formation and endurance during treadmill exercise [73].

Transgenic overexpression of the transcription factor Nr4a1 in skeletal muscle in mice leads to increased glycogen content, increased oxidative metabolism with increased fatty acid consumption, as well as enhanced respiratory capacity of isolated mitochondria, improved muscle contraction ex vivo, and heightened cold tolerance and grip strength in vivo [15]. Furthermore, it leads to upregulation of insulin-like growth factor 1 (Igf1) transcription and downregulation of myostatin transcription, as well as increased muscle fiber size [91]. Transgenic overexpression of Nr4a2 in skeletal muscle leads to increased transcription of Glut4 and G6pd, enhanced glycogen storage, enhanced glucose uptake and tolerance under a high fat diet, as well as to increased exercise performance [1]. Muscle-specific transgenic overexpression of Nr4a3 leads to an increased number of type 2 muscle fibers and several behavioral and physiological effects such as improved glucose tolerance, improved oxygen consumption, and enhanced running endurance [70]. At the cellular level, Nr4a3 overexpression results in enhanced myoglobin expression, mitochondrial density, oxidative enzymes, and electron transport chain complex proteins, as well as increases in mRNA transcripts and proteins for IIA and IIX myosin heavy chain and decreases for type IIB myosin. Overexpression of Junb proto-oncogene (Junb) via transfection into muscles in adult mice leads to muscle hypertrophy as determined by an increased cross-sectional area and to increased protein synthesis, as well as protection against muscle atrophy after denervation [77]. Bdnf overexpression in skeletal muscle in vivo leads to induction of a glycolytic fiber type and a fast muscle-type gene program, while its muscle-specific knockout leads to enhanced fatigue resistance and improved exercise capacity [20]. Muscle-specific, transgenic Pgc1a overexpression in mice enhances mitochondrial function, peak oxidative capacity, and exercise capacity [12]. Another study reported similar findings, with muscle-specific Pgc1a-b overexpression increasing mitochondrial gene expression and biogenesis, glycogen content, lipid oxidation during exercise, and exercise performance [88].

## Methods for enhancing adaptive transcription

The results above demonstrate that targeting adaptive transcription is a viable strategy to improve physiological function, increase resilience to stressors, and counteract aging-related physiological decline. One critical challenge to leveraging these insights for impacting human health is how to influence adaptive transcription.

### Small molecule drugs and natural compounds

One potential way to enhance adaptive transcription is by application of single molecules, which can include different classes such as synthetic small-molecule compounds, peptides, or naturally occurring compounds. Previous work has explored the design of CREB potentiators for memory improvement [101]. Crebinostat, a compound that enhances CREB signaling via histone deacetylase (HDAC) inhibition, increases Egr1 induction, synaptogenesis, and memory in mice [24]. Treatment with the HDAC inhibitor trichostatin A increases memory and synaptic plasticity in a CREB-dependent manner [92]. HDAC inhibition via oral administration of suberoylanilide hydroxamic acid (vorinostat) restores spatial memory performance in a mouse model of Alzheimer’s disease [8] and HDAC4 inhibition prevents denervation atrophy of muscles in mice [51]. In humans, HDAC inhibitors have transitioned into the clinic [49], demonstrating that in principle, targeting HDACs can lead to physiological effects with an acceptable side-effect profile. Inhibition of PDE4 via rolipram leads to CREB induction, Arc upregulation, and memory improvements in rats [62], enhances synaptic plasticity and memory in mice [4], improves memory in aged mice [3], and rescues memory performance in Alzheimer’s disease mouse models [29]. The putative Nr4a1 activator amodiaquine improves glucose tolerance and resistance to obesity in mice [1].

### Gene and cell therapy

As AAV vectors are gaining traction in clinical settings [98], one potential way to directly increase the activity of adaptive transcription programs is via virus-mediated gene delivery. In many of the studies cited above, genes were delivered to their locus of action through viral vectors including AAVs. This hence opens the possibility of viral-mediated overexpression of proteins such as CREB to enhance neuronal plasticity and resilience. An interesting alternative method to induce adaptive transcription genes in the brain is via peripheral gene delivery. The authors of one study delivered the gene for the myokine fibronectin type III domain-containing protein 5 (FNDC5) to the liver via AAV and this treatment induced several IEGs in the brain [100]. Skeletal muscle overexpression of the transcription factor TFEB increases exercise endurance in young animals and enhances cognitive function in aged animals [59]. Yet another way to influence adaptive transcription could be through cell therapeutic approaches such as cell transplantation. Previous work has explored in theory how genomically modified cells could be used to influence tissue function in goal-directed ways [41, 43]. As experimental work has shown that stem cells with inducible gene expression systems can be constructed [58, 76, 108] and that adaptive transcription factors can be induced by systemically released molecules (e.g., IEG induction in the brain through FNDC5 [100]), a novel therapeutic concept could aim to deliver a relatively small number of cells to control body-wide, temporally restricted induction of adaptive transcription. Additionally, with the advent of RNA delivery technologies [69], RNA-based expression of transcription factors might be able to achieve induction of adaptive gene programs.

### Dynamics and tissue specificity

The intervention methods discussed above have different advantages and disadvantages. A central consideration with targeting adaptive transcription relates to the underlying dynamics. Under physiological conditions, adaptive gene programs are activated within minutes and are usually active for several hours before being shut off. To mimic these dynamics, pharmacological approaches and inducible gene expression methods might be better suited than gene expression from constitutively active promoters. For instance, inducible Egr1 overexpression in the brain via the tetracycline system has been employed for memory enhancement [71]. Prolonged unphysiological expression patterns might lead to maladaptation (see below). With regard to gene delivery via AAV-based vectors, previous work has shown that a tetracycline-dependent gene expression system can be fully encoded in a single vector, which could enable temporal specificity [17].

Another concern relates to tissue and cell type specificity. As reviewed previously, complex physiological stimulation patterns elicit adaptive transcription in several tissues at once [42]. As such, the generally broader tissue distribution of pharmacological approaches in comparison to localized gene delivery could be an advantage as adaptive gene programs could potentially be boosted across the whole body. On the other hand, if restricted induction would be necessary, i.e., if boosting adaptive transcription could disproportionally raise maladaptation in certain organs (see below), tissue specificity and hence AAV-based delivery would be preferred. Furthermore, the same transcription factors can control different target gene programs with different downstream effects in different cell types which is important with regard to cell tropism in AAV-based deliveries (e.g., Npas4 controls different gene programs in excitatory vs. inhibitory neurons with resulting differential effects on excitatory and inhibitory input onto cells [84]). Similarly, CREB regulates different target gene programs in different human tissues through the differential recruitment of coactivators [106].

## Targeting adaptive transcription is well-suited to enhance physiological function

With the above results in mind, we can now explore why targeting adaptive transcription would be a particularly useful strategy to improve organism function and counteract aging-related decline.

First, at the cellular level, adaptive transcription components represent central signaling nodes that control and coordinate complex downstream molecular cascades. Targeting crucial regulator molecules such as CREB allows for widespread and coordinated cell changes, thus leveraging “pre-configured” natural and effective cellular adaptation programs. Current biomedicine is well-equipped to impact single or a few molecules and targeting adaptive transcription might hence combine the conceptually straightforward and technically feasible approach of single-molecule targeting with the effectiveness of complex natural adaptation programs.

The second major benefit stems from the fact that performance improvements and anti-aging efforts usually require multi-tissue coordination. Increases in athletic capacity such as endurance running require adaptations in several types of cells and tissues such as muscle, nervous, and fat tissue. Similarly, aging-induced decline of body function happens in several tissues at once (decreased cognition, decreased muscle function, dysfunctional metabolic maintenance). Since adaptive transcriptional programs are active in all major organ systems (reviewed in [44]), they represent particularly well-suited targets to facilitate body-wide complex remodeling via conceptually simple means.

A third advantage is that adaptive transcription is physiologically active on the scale of hours while its effects (i.e., remodeling of cells and cell-networks) last from hours to days to potentially years (e.g., in long-term memory formation in humans). This means that relatively short-lived interventions (e.g., application of a pharmacological agent) to boost adaptive transcription could potentially be translated into long-lasting benefits. This could enable a treatment paradigm in which, during curative treatment periods, an organism engages its adaptation mechanisms, followed by longer periods of improved function without the need for continuous therapeutic intervention.

## Caveats and open questions

There are several caveats and open questions associated with targeting adaptive transcriptional programs for health improvement.

First, adaptive transcription has a crucial role in many maladaptive processes, including oncogenesis, autoimmunity, addiction, and cardiovascular diseases, as previously reviewed [44]. The conundrum that adaptation processes also implement maladaptation and can thus harm the organism has been termed the *adaptation-maladaptation dilemma* [45, 46]. Overactivating adaptive transcription, especially with unnatural dynamics such as in constitutive overexpression, might thus lead to substantial negative side-effects and drive pathology. CREB overexpression for instance has been shown to induce an epileptic phenotype [50] and can, depending on the expression dynamics, interfere with spatial information recall [96]. Additionally, CREB has an important role in the progression of central nervous system cancers [21] which means that potential oncogenic effects must be taken into consideration during intervention design. Overexpression of AC1 leads to enhanced CREB signaling and slower memory extinction [99]. Npas4 has been shown to be involved in neuroprotection [105] but also positively regulates the response to drugs of abuse [47]. Thus, one set of challenges revolves around the precise temporal control of adaptive transcription in vivo to avoid maladaptation*.* As has been previously explored in the context of aging, one way to achieve this might be via careful selection of the stimulation protocol, as different dynamics of the same signaling molecules can have different downstream effects (e.g., sustained ERK activation drives differentiation while transient activation drives proliferation [56]).

Previous work has also explored how adaptive cellular reprogramming might necessitate recovery periods as it uses cellular resources and induces certain types of molecular damage [42]. For instance, adaptive gene induction has been reported to require DNA strand breaks [52]. Sleep has been linked to DNA strand break accumulation during wakefulness [103], as well as enhanced DNA strand break repair [104], thus possibly hinting at the necessity for recovery periods caused by adaptive gene activation.

With regard to aging, time-dependent changes in the levels or activity of adaptive transcription mediators and their co-factors have to be considered. For instance, the levels of the CREB co-factor CREB binding protein (CBP), a histone acetyltransferase, decrease in the brain with age [18] which could lead to difficulties in targeting this pathway for health improvement. Similarly, aging is associated with changes in stimulus-induced adaptive transcription in the immune system [57] which could mean that targeting these programs might have unintended side-effects.

Another caveat is that most of the studies discussed here that directly demonstrate physiological improvement through manipulations of adaptive transcription were performed in rodents. Previous results have highlighted important differences in transcriptomic programs between human and mouse cells such as for instance that human iPSC-derived neurons display several unique adaptive gene induction patterns when compared to mouse cells [2, 75]. Measuring and impacting human-specific adaptive transcriptome changes might be required for optimal physiological enhancement.

An important task going forward will be to integrate adaptive transcription-based improvement strategies into multi-scale intervention concepts. Mere overactivation of single molecules by itself is unlikely to increase physiological function in complex tasks or lead to a protection against age-related multi-organ decline. One way forward might hence be to study and develop adaptive transcription boosters that will be applied in conjunction with complex stimulation patterns at the whole organism level (e.g., exercise and environmental enrichment) and/or at the organ level (e.g., bioelectric stimulation of whole organs such as ECT in the brain which in animals induces adaptive transcription [65, 90], cellular plasticity [85], and behavioral improvements [36]), as previously suggested [44]. Adaptive transcription-based enhancement can thus be an important part of systems physiology approaches by facilitating and boosting complex phenotypic state transitions through single-molecule targeting.

## Conclusions

One major way of controlling cellular adaptation processes in animals is adaptive transcription and under physiological conditions these gene programs control a wide variety of plasticity mechanisms including learning and memory and exercise-dependent muscle remodeling. As the present work explores, directly overexpressing or activating several adaptive transcription components leads to enhanced cognition and exercise function in adult animals, as well as to protective effects against aging-related decline of physiological function. Several interventions can potentially enhance adaptive transcription and provide beneficial physiological effects ranging from pharmaceutical compounds to gene transfer technologies. In the future, directed efforts will be needed to target adaptive transcription programs in humans and integrate them into multi-scale systems physiology enhancement approaches.

## Data Availability

No datasets were generated or analysed during the current study.
